# Use of NT-proBNP in weaning from mechanical ventilation

**DOI:** 10.1186/cc9582

**Published:** 2011-03-11

**Authors:** A Martini, B Benedetti, N Menestrina, E Polati, E Bresadola, L Gottin

**Affiliations:** 1 University Hospital, Verona, Italy

## Introduction

Our objective is to evaluate the role of the levels of B-type natriuretic peptide (BNP), released in response to increased wall tension, as a predictor of weaning failure.

## Methods

We enrolled 98 patients, admitted to the ICU for acute respiratory failure, who underwent mechanical ventilation and were considered ready for a weaning trial. Patients were divided by means of echocardiographic criteria into four groups according to the severity of heart dysfunction: Group 1: normal left and right ventricular function and absence of relevant valvulopathy; Group 2: mild left systolic ventricular dysfunction, ejection fraction >40%, mild valvulopathy, diastolic dysfunction >II; Group 3: moderate to severe left systolic ventricular dysfunction, ejection fraction <40%; and Group 4: severe right ventricular dysfunction: ventricular volumes R/L >0.6, arterial pulmonary pressure >30 mmHg. Plasma NT-proBNP was measured just before (BNP 1) and at the end (BNP 2) of the weaning trial in all patients. Patients who passed the weaning test were finally extubated. Extubation was considered failed if the patient required reintubation within 48 hours. We compared plasma BNP concentrations in the different groups with Mann-Whitney or chi-square tests and we considered also ΔBNP (BNP 2 - BNP1) and %Variation (Δ/BNP1).

## Results

In the whole sample NT-proBNP levels were not significantly different in patients who had a positive weaning and in those who failed it. ΔBNP and %Variation were higher (*P *< 0.001) in patients who failed the test than in patients who passed the test. In Group 1 a higher ΔBNP, and in Group 2 a higher ΔBNP and %Variation, were correlated with weaning failure. In Group 4, instead, the plasma BNP concentration decreased during the weaning test. ROC curve analysis was performed to assess ΔBNP and %Variation's ability to discriminate between patients who had a positive weaning and those who failed. In Group 1 the area under the ROC curve values were 0.88 for ΔBNP and 0.94 for %Variation. In Group 2 the area under the ROC curve values were 0.64 for ΔBNP and 0.86 for %Variation.

## Conclusions

Recent papers evaluated the role of BNP in patients who had undergone mechanical ventilation. In our population ΔBNP and %Variation before and after the weaning test are more reliable than NT-proBNP levels to detect extubation failure in patients with mild cardiopathy or without relevant cardiopathy. In patients with severe cardiopathy because of the complexity of clinical pattern, NT-pro-BNP cannot be used as a predictive marker of extubation failure.

